# Report of the Meeting of the Provincial Medical and Surgical Association

**Published:** 1836-10

**Authors:** 


### PROVINCIAL MEDICAL AND SURGICAL ASSOCIATION.

The fifth anniversary meeting of this interesting and important Association has been held this year in Manchester. It commenced on Wednesday, July 20th, at one o'clock, when the Members of the Council assembled in the Council Room of the Royal Institution, to transact the business of the Association, and to arrange the plan of the public business for that and the succeeding day. Dr. Kidd, Professor of Medicine, &c. at the University of Oxford, was voted to the chair, and among the Members of Council present were, Dr. Hastings, of Worcester, principal Secretary of the Association, Dr. Jeffreys and Dr. Scott, of Liverpool, Dr. Barlow, of Bath, Mr. Hebb (Mayor) of Worcester, Dr. Brown, of Sunderland, Dr. Conolly, of Warwick, Dr. Holme, of Manchester, Dr. J. L. Bardsley, of Manchester, Dr. Streeten, of Worcester, Mr. Griffiths (Mayor) of Hereford, Dr. J. Johnstone, of Birmingham, Mr. Ransome, Mr. Jordan, and Mr. Turner, of Manchester, Dr. Shaw, formerly of Manchester, Mr. Flint, of Stockport, and Mr. Crosse, of Norwich.

In the evening, at eight o'clock, the members of the Association assembled in the room of the Royal Institution usually devoted to the Choral Society. The attendance was respectable and numerous, upwards of a hundred medical men being present. Among them, in addition to those we have named, were several of the leading practitioners of Manchester, and many strangers from neighbouring towns. On the motion of Professor Kidd, seconded by Dr. Hastings, Dr. Holme, President for the year, was called to the chair. He opened the proceedings with a short address, in which he intimated the advantage which gentlemen would derive, if, after the business of the Association was at an end, they could spend a day or two in the investigation of the state of society in Manchester, particularly among the working classes, and would enquire into the influence which the introduction of manufactures and the hours of labour have upon the health and comforts of the working poor. Every facility would, he was sure, be afforded by the liberality of the manufacturers. He would especially call the attention of those gentlemen who resided in districts purely agricultural to this subject. They were all aware that considerable diversity of opinion prevailed regarding it, but he was sure gentlemen belonging to an honorable profession would approach the question with unbiassed minds, and see for themselves how far the representations which had gone forth to the world were consistent with truth. Dr. Hastings next read the report. It announced an accession of one hundred new members, during the year, and called attention to the fact that a similar Association had been formed in the Eastern counties, the members of which, anxious to join the Parent Institution, had sent a deputation of six of its members to negociate an alliance. The income of the year is stated to have been, including the balance in hand, 705*l.*, the outlay 498*l.* 1*s.* 7*d.*, leaving a balance of 205*l.* It mentioned the existence of considerable arrears, and suggested that it might be desirable to appoint paid collectors in certain districts. It regretted the omission from the last volume of Transactions of many contributions worthy of publication, which were inevitably deferred to a future volume, regretted that the large Hospitals and Infirmaries had forwarded so few statistical observations and reports of cases to the Association, and concluded by urgently inviting the attention of members to the investigation of epidemic diseases, which was one of the original and principal designs of the Association. The accumulation of numerous facts, a register of the rise, progress and decline of epidemics in each district, with a careful minute of dates, and a noting of the condition of the atmosphere, its barometric, thermometric, and hydrometric state, was the thing needed, the object being, not to build a theory, but to record facts from which useful deductions may hereafter be made. The registry, complete to the last day of May, should be forwarded to the nearest member of the Council, and by him transmitted to the Secretaries.

On the motion of Dr. Kidd, seconded by Dr. J. Johnstone, the report was adopted. Mr. Tudor, of Bath, moved the appointment of a Committee, to consider whether it was desirable, and if so on what terms, to form a junction with the Eastern Association, the Committee consisting of Messrs. Barlow, Hebb, Streeten, Kidd, Jordan, and Brown. The motion, being seconded by Dr. Jeffreys, was carried. On the motion of Dr. Scott, seconded by Mr. Ransome, thanks were voted to Dr. Kidd for his services during the year as President, with a high eulogium on the manner in which the Association had last year been entertained at Oxford.

Dr. Barlow, of Bath, proposed the thanks of the meeting to Dr. Hastings and Mr. Sheppard, the Secretaries, with a request to them to continue their services. He said it would be a waste of the time of the meeting to utter a single expression in praise of the motion, but he might say that the resolution could not have been placed with more propriety in the hands of any other person, because it happened that his (Dr. Barlow's) knowledge of the Association preceded (to use an Hibernicism) its formation. When the first conception of it glanced across the mind of Dr. Hastings, he (Dr. B.)

was the earliest friend he consulted, and he had his cordial encouragement to persevere in the noble career he had opened. He never doubted of success, and every body would now perceive the justice of his anticipations.

Dr. Conolly, of Warwick, moved a vote of thanks to the Council and their re-appointment, with the following additional members: Dr. John Alexander, Dr. Lyon, Mr. Hunt, Mr. Noble, Mr. Boutflower, Mr. Jordan, of Manchester, Dr. Kay of Clifton, Dr. Turner of Stockport, Dr. Haviland, Professor of Medicine in the University of Cambridge, Dr. Williamson of Leeds, Mr. Wilson of Gloucester, Dr. Vassall of Aberystwith, Dr. Travers Cox of Yarmouth, Dr. Hudson of Navan, Ireland, Mr. John Needham of Leicester.

Dr. Goldie, of Shrewsbury, proposed the thanks of the meeting to Mr. Hebb and Dr. Clark, for the trouble they had taken in communicating with foreign correspondents, aud requested Dr. Clark to convey thanks to Dr. Nassé, of Bonn, for his interesting paper (on the present state of anatomical knowledge in Germany.) Mr. Turner, of Manchester, after paying a warm tribute to his friend, proposed that Dr. James L. Bardsley should be requested to prepare the retrospective address for the annual meeting in 1837. Dr. Streeten, of Worcester, seconded the motion, remarking, that highly as his townsmen might estimate the merits of Dr. Bardsley, they did not estimate them more highly than he. The motion was carried with applause, and was briefly acknowledged by Dr. Bardsley. Dr. Barlow next read a report from the Committee appointed to prepare the rules and obtain subscriptions for the Benevolent Fund. He read the fundamental regulations, which placed the fund simply on the basis of a Benevolent Society, not of an Insurance Association or a Benefit Club. The income up to June has been—Donations announced, 199*l.* 7*s.*, (received, 106*l.* 5*s.*); Subscriptions announced, 49*l.* 7*s.*, (received 22*l.* 1*s.*) making the whole amount announced, 248*l.* 14*s.*, and received, 128*l.* 6*s.* The disbursements have been 11*l.* 15*s.* 2*d.*, leaving a balance in hand of 116*l.* 10s. 9*d.*

Dr. Kidd then read to the meeting a paper, consisting of remarks on the anatomical and physiological works of Galen. It set out by expressing an opinion, contrary to the accepted doctrine, that Galen drew his descriptions of anatomy from the human subject, and stated that nearly all the anatomical terms he used were still in use, and for the same purposes to which he applied them. He observed that, to give an account of Galen's system of osteology, would be almost like reading a treatise of any modern writer on the subject. He instanced, as a proof of the philosophical spirit in which Galen pursued his profession, that a man having lost the use of the extremities of his fingers, and vainly sought relief from local remedies, Galen enquired if he had ever received an injury of the spine, and learning that in early life he had, he directed his remedies to the region of the injured spine, and so restored the use of the fingers. Galen maintained that the reasoning power resided in the brain, and that physical structure was indicative of the power of the brain ; but he declared that on all occasions, when speaking of the moral actions of men as connected with their physical structure, he only spoke of their innate propensities, not of their conduct as the result of education.

Mr. Ayre, of Leeds, exhibited an apparatus for treating distortion of the spine. It was very simple, its object being to keep the patient quite fixed in an horizontal position, without, however, inflicting the least personal inconvenience. His course of practice, he said, had been always to obtain a straightening of the spine, then to impart bodily strength to the patient, and thus to encourage a healthy deposit for the creation of bone in the weakened part. He exhibited drawings of cases.

The members of the Association met again on Thursday, at twelve o'clock, the attendance being again numerous. Dr. Holme having taken the chair, Dr. Hastings drew attention to the fact that reports on various subjects, which gentlemen had at former meetings been deputed to draw up, had not yet been received.

Mr. Crosse then read the retrospective address for the past year, which was extremely interesting. As it was unanimously decided that this should be printed, we shall have another opportunity of noticing it. It was received with much applause.

Dr. Kidd read the report of the Committee appointed to confer with the deputation from the Eastern Association on the subject of a junction: the report recommended a delay for a year, the present terms proposed not being such as could be adopted, whilst it was hoped that by a modification of them the object would be accomplished. On the motion of Mr. Wilson, of Manchester, seconded by Mr. Bagnall of Chester, the report was ordered to be printed and its proposal adopted, and Dr. Hastings expressed the obligations of the Association to the gentlemen of the East for travelling so far to effect this desirable object—a sentiment which was afterwards embodied in a motion, and being proposed by Dr. Kidd, and seconded by Dr. Barlow, was adopted. On the motion of Dr. Barnes, of Carlisle, seconded by Dr. Lyon, of Manchester, Cheltenham was fixed on for the next year's meeting, and Dr. Boisragon was chosen President elect.

Dr. Knight, of Sheffield, seconded by Mr. Barnett, of Stourport, proposed the formation of a Committee to investigate and report upon the various modes of extending medical relief to the poor, independent of parochial relief, the Committee to consist of Drs. Forbes, Walker, Conolly, and Barlow, Messrs. Smith, H. W. Rumsey and Nankivill. Mr. Smith, of Southam, brought forward a paper embracing a comparison of the self-supporting Dispensaries, (of which he is the founder,) with other benevolent institutions and clubs. It was not read, but will appear in the Transactions. Dr. Barlow, seconded by Dr. Black, of Bolton, proposed a vote of thanks to the managers of the Royal Institution, and the other public institutions in Manchester, for their courtesy. On the proposal of Dr. Walker, a renewed tender of thanks was made to the Secretaries, and the proceedings terminated in a vote of cordial thanks to Dr. Holme, which was proposed by Dr. Kidd and seconded by Dr. Conolly. The latter gentleman observed that it was not the number of their members, nor the extent of country over which they spread their influence, nor the bulk of their transactions, nor the number of persons attending these delightful annual assemblages, that constituted the most honorable feature of their Association. It would always derive its greatest recommendation to the public and its greatest power of doing good—without which all assemblages were useless—from being honoured with the countenance and cooperation of those who were illustrious for the talent they displayed, the industry they exercised, for their great attainments, and, above all, for the virtue of their character. He said thus much after the very short speech of Professor Kidd, because it gave him the opportunity of alluding to that to which Dr. Kidd could not allude, namely, the circumstance that they had met for successive years under the auspices of Dr. Johnstone, Dr. Carrick, Dr. J. Johnstone, Dr. Kidd, and Dr. Holme. The mention of these names would remind the profession of all the exalted qualities that adorned it, and it was this among the thousand recommendations which made every man proud of being a member of the Association. In returning, therefore, to Dr. Holme their best thanks for the honour of his Presidency, they were returning a debt of gratitude delightful to owe and delightful to pay. It was delightful, too, to witness—and, for himself, he could never sufficiently express himself on this subject—to witness in those whose names were most honoured in the profession, and whom they all should most desire to see honoured, a simplicity of manner and all those indications of amiable character which were so often found in the profession. The Association must always prosper, so long as it numbered among its Presidents those who resembled Dr. Holme.

In the evening the members of the Association, to the number of nearly two hundred, dined together with a few guests, among whom was the illustrious and venerable Dr. Dalton. The chair was occupied by Dr. Holme, and the vice-chairs by Mr. Turner, Mr. Wilson, and Dr. J. L. Bardsley.

Many toasts were drunk and many excellent speeches delivered, but we can only find room for a brief outline of that of Dr. Hastings, on his health being drunk in connexion with the "Provincial Association;" and that of Mr. Turner on his name being coupled with "Provincial Schools."

Dr. Hastings said he could not attempt adequately to express the feelings with which he was overcome when he considered the very distinguished manner in which he had been treated during the whole of this great, influential, and scientific meeting in Manchester, by every member of the Association with whom he had in any way come in contact. The very proudest periods of his life were those he had spent with the members of the Association, and to his dying day he should consider it one of his highest distinctions to be regarded one of the founders—though a very unworthy one—of the great, flourishing, and extraordinary Association which, he trusted, would carry down to posterity a memorial of the exertions which were now making for the advancement of medical knowledge, and unite together in one firm bond the now disjointed members of the medical profession in the provinces. But a very few years ago they were in every sense of the word a disjointed set of people. A town existed here and there with a distinguished member of the profession rising up in it, but like the little states of England in early times, they were disconnected as a class—little rivalries and jealousies kept them asunder, and as a body they made no progress. He took no praise to himself, because by this Association the evil was likely to be abated, and the whole profession in the provinces likely to become linked together in one firm bond of mutual co-operation and reciprocal good offices. The condition of things was favorable for receiving the proposal at the very moment it was made, and thus it had happened that the Association had risen to an eminence which in the first workings of the idea in his own mind he never could have foreseen. It could not indeed have been supposed, looking to the state of the provinces eight years ago, that after the lapse of a few years an Association should branch forth and extend itself, as this had done, over every part of England. It was indeed a proud day for them, when they met in Oxford under the auspices of the learned Professor, and when the most learned of the land and the most distinguished of the University came forward to do homage to their Association. They were, however, equally fortunate on this occasion in meeting in this sub-metropolis of the country, the centre of the cotton manufacture, which had tended so much to increase the benefits of modern civilization. In Manchester they had an instance, such as was not afforded in any country in the world, of the intimate connexion between science and commerce—and no where had that connexion been carried forward with so much advantage to the community as in this great city. This was the first provincial town in which the memoirs of any scientific society were ever published, and he was sure every one would agree with him in thinking that at the present day Manchester contained many eminent and worthy successors of Percival, Ferriar, and White.

Mr. Turner said, nothing could afford him more pleasure than the circumstance of having this name associated with the toast which had just been given; for he did not hesitate to declare that there was no subject nearer his heart, and none he was more anxious to accomplish than the establishment of Provincial Schools, from a conviction that they were destined to have an important effect on the future education of medical men. It was not remarkable that "The Success of Provincial Schools" should be among the appointed toasts of that evening; but as there existed a difference of opinion as to the practicability and efficiency of provincial medical education, it could not fail to be extremely gratifying to the provincial teachers there assembled, to find that so large a majority of the intelligent and influential members of the profession should be advocates for it; for what other interpretation could be put upon the enthusiasm with which they had received the toast proposed from the chair? It was not necessary for him to dwell upon the rise and progress of such establishments. The necessity for them was created by the growing intelligence of the times—times when education was giving such an impulse to all classes of society, as would tend to elevate the character of man, as a social and intelligent being. Under these circumstances, was it not a duty incumbent upon them to improve the condition of the medical student, so as to enable him to keep pace with the other branches of society, and to maintain that dignity and science which belonged to their profession, which might otherwise be swept away in the stream of knowledge now rushing with such tremendous impetuosity through all the walks of life. It was not possible to sum up in a few words the advantages which would be sure to accrue from Provincial Medical Schools; but viewing them in their more humble pretensions, if they have the effect of directing the mind of youth into the proper channels for information—of preventing the assiduous student from languishing for want of opportunity of acquiring knowledge—they surely were objects of interest, and well deserving of the support of the moralist, the philanthropist, and every well-wisher to science and the medical profession. He concluded by observing that proofs were not wanting, that the education given in Provincial Establishments was good and true; and he would venture to appeal to the examining bodies of the profession, and ask, whether recent experience has not tended to confirm the dictum, both publicly and privately pronounced, that nothing has contributed so much to enhance the dignity of the profession, by giving better education to its members, as the establishment of Provincial Schools.

---

SOUTHERN BRANCH OF THE PROVINCIAL MEDICAL AND SURGICAL ASSOCIATION.

